# The impact of technological innovation on transport carbon emission efficiency in China: Spillover effect or siphon effect?

**DOI:** 10.3389/fpubh.2022.1028501

**Published:** 2022-10-04

**Authors:** Qifei Ma, Peng Jia, Haibo Kuang

**Affiliations:** ^1^School of Maritime Economics and Management, Dalian Maritime University, Dalian, China; ^2^Collaborative Innovation Center for Transport Studies, Dalian Maritime University, Dalian, China

**Keywords:** technological innovation, carbon emission efficiency, transport industry, spatial effect, government intervention, mechanism analysis

## Abstract

It is currently unknown whether technological innovation will have spillover or siphon effects on transport carbon emission efficiency (TCEE). Therefore, this paper creates a spatial econometric model to explore the spatial effect of technological innovation on TCEE. Taking 30 provinces in China as examples, we find that the TCEE and the technical innovation index have similar evolution characteristics (numerical value grows, the gap widens), and that both have a spatial distribution that decreases from the eastern coast to the western inland. Further research reveals that TCEE has a considerable siphon effects in China. The siphon effect gets stronger the higher the TCEE. Although technology innovation has the potential to improve TCEE in local province, the siphon effect hinders TCEE improvement in surrounding provinces. Furthermore, heterogeneity research reveals that excessive government intervention will inhibit the promotion of technological innovation on TCEE. Greater levels of government intervention in the middle and western regions than in the eastern region have more obvious inhibitory impacts. The results demonstrate that economic growth and transport structure have played a mediating role in the process of technological innovation promoting TCEE. Regional collaboration and less local protectionism can help the government achieve the dual goals of technological innovation development and TCEE promotion.

## Introduction

Transportation is a basic and leading industry to support the development of social economy. It is also an important field of energy consumption and carbon dioxide emissions (1). According to International Energy Agency (2), China's carbon emissions accounted for 31% of the global carbon emissions in 2020, while transportation accounted for about 10% of the national carbon emissions, making it the third largest carbon dioxide source after industry and construction (3). Carbon emissions from transport sector will continue to rise in the future as people's living standards rise (4). China is facing enormous pressure to reduce emissions.

In order to achieve the emission reduction targets of the transportation industry, the Chinese government has issued a series of policies and laws. In 2019, the State Council issued the Program of Building National Strength in Transportation, which clearly pointed out that it is necessary to promote the transformation of transportation development from pursuing speed and scale to focusing on quality and efficiency, shift attention from independent development to integrated development, and promote innovation instead of relying on traditional elements (5). The report of the Communist Party of China's 19th National Congress pointed out that scientific and technological innovation is not only a critical factor in achieving the carbon emission reduction target and developing a low-carbon economy, but also an inherent requirement for China's high-quality economic development. It has become the main driving force of carbon emission reduction in China, and the improvement of carbon emission efficiency has also forced the government and enterprises to carry out scientific and technological innovation to varying degrees (6).

At present, it is still unclear how technological innovation will affect the transport carbon emission efficiency. Some researchers believe that technological innovation may reduce carbon emission efficiency, because it may require more energy consumption, which will lead to more carbon emissions (7, 8). However, more researchers are coming to the conclusion that technological innovation can improve carbon emission efficiency by developing new techniques to reduce carbon emissions and improving traditional measures of pollutant emission reduction (9–12). Furthermore, according to Karacay (13), capital, labor and other elements of production tend to flow to regions with advanced technology, which may reduce the carbon emission reduction capacity of regions with backward technology. This statement also applies to China. Although China's science and technology has advanced quickly over the past 40 years, there is still a significant imbalance between regions, and the competition is fierce (14). Over-concentration of production factors in sophisticated regions will reduce the overall resource allocation efficiency when resources are constrained. The technological development gap would widen due to insufficient growth drivers and incentives in neighboring provinces, ultimately lowering carbon emission efficiency (15).

In addition, evidence indicates that technological innovation has obvious spatial dependence (16). The advancement of science and technology in one region affects not only local economic activities and carbon emissions, but also the production and living activities of other regions through information transmission and factor flow, so impacting carbon emission efficiency (17, 18). Therefore, spatial effect is a critical consideration when assessing the influence of technical innovation on carbon efficiency. Most studies, however, have overlooked it. Although some scholars have taken into account the spatial effect, most of them have neglected the influence of spatial weights on the spatial correlation of carbon emission efficiency (19). The existing literature mainly adopts a symmetrical weighting scheme based on the adjacency or distance principle, and the mutual influence between the two evaluation units is consistent by default (20, 21). In fact, the transportation network transcends geographical boundaries and distance constraints, enabling people and goods in non-adjacent regions to be transported over long distances, which may lead to the asymmetry of the interaction between the two provinces, and thus lead to inaccurate measurement results.

Based on the above analysis, there is no uniform answer to the impact of technological innovation on TCEE at present, we think it is necessary to find out the spatial relationship between technological innovation and TCEE, which will help to find out the driving mechanism of TCEE and provide a new path for carbon emission reduction. Our research has made contributions to the existing literature. Firstly, the theoretical foundation has a certain frontier. According to the concept of green development and the strategic requirements of a strong transportation country, we have separately constructed the evaluation framework of transportation carbon emission efficiency and science and technology innovation index, and discussed the spatial relationship between them, thus organically integrating the green development of transportation industry, environmental constraints and science and technology innovation. The research results enrich the theoretical framework of carbon emission efficiency of transportation and improve the development theory of transportation emission reduction path.

Secondly, the research perspective innovation. Based on the spatial theory, we use spatial pattern statistics and testing methods to measure the spatio-temporal characteristics and spatial relationship of China's transportation carbon emission efficiency and science and technology innovation index from the macro level. We also use the principles of proximity, distance and reciprocal of economic distance to construct three spatial weight matrices, which prove the rationality of asymmetric spatial weights in judging TCEE spatial relations. This is helpful to clarify the growth mechanism of carbon emission efficiency of transportation, and provides a new idea for speeding up carbon emission reduction of transportation.

Thirdly, the research results are worth popularizing. On the basis of identifying the spatial characteristics of China's transportation carbon emission efficiency, the spatial effect of scientific and technological innovation on transportation carbon emission efficiency, the heterogeneity and transmission mechanism of government intervention are measured by spatial econometric model, and targeted and differentiated strategies for improving transportation carbon emission efficiency are put forward. Our conclusion is helpful to fully understand the spatial effect of scientific and technological innovation on carbon emission efficiency of transportation, and have important practical value in assisting the transportation industry to cope with carbon emission peak and carbon neutrality.

## Literature review

The carbon emission efficiency is very important for the building green transportation. Transport sector is one of the major carbon emitters. Improving the carbon emission efficiency of the transport sector is the essential courses to build green transportation and realize carbon neutrality. Technological innovation is an important carrier and breakthrough to drive the transformation of energy structure and improve energy efficiency, which can affect carbon emission efficiency to a large extent. Therefore, we will review relevant literature from two aspects: carbon emission efficiency and the influence of technological innovation on carbon emission efficiency.

Many scholars have conducted relevant research on carbon emission efficiency, with a particular focus on the following aspects: carbon emission efficiency measurement (22, 23), spatial effect (24, 25), and driving factors (26, 27). Single-factor method was first applied to measure carbon emission efficiency due to its simple operation (28–30). Subsequently, some scholars began to use the total-factor evaluation method, such as data envelopment analysis (DEA), to measure the TCEE (31, 32). For example, Cui and Li (33) employed the virtual frontier data envelopment analysis model to evaluate the TCEE in 15 countries and used the Tobit regression model to identify the major contributing factors. Ren et al. (34) established a DEA model with radial opportunity constraints to calculate the TCEE of China. Park et al. (35) used the SBM model to assess the environmental efficiency of the transport industry in the United States from 2004 to 2012, and estimated the carbon emission reduction potential of 50 U.S. states. Omrani et al. (36) rank the operation efficiency of the transport departments in Iran's provinces using the cooperative game and cross-efficiency technique.

In addition, some scholars have discussed the spatial heterogeneity of carbon emission efficiency, but neglected the influence of asymmetric spatial weights (2, 37–39). Previous research has primarily used symmetric spatial weight matrices, such as the adjacency and distance matrices, in which the mutual impact between the two evaluation units is consistent by default (19). However, this does not fully reflect the mutual influence of geographical elements in different evaluation units. For example, due to varying levels of economic development, the impact of province i on j is often different from that of province j on i. Therefore, setting the spatial weight among the evaluation units as an asymmetric weight in the application process can more effectively reflect the spatial heterogeneity of geographical elements (40, 41). Besides, scholars also use exponential decomposition (42, 43), input-output (31, 44) and econometric models (45, 46) to study the driving mechanism of carbon efficiency. Unlike the index decomposition, input-output and the traditional econometric regression model, the spatial econometric model can take account of the spatial factors, and gradually becomes the mainstream method for investigating the elements that influence carbon emission efficiency (47).

At present, there is no unified assessment standard for the index of technological innovation, and academic circles hold different views on the impact of technological innovation on carbon efficiency. Many academics argue that technical innovation can promote the promotion and utilization of new energy and the improvement of energy use efficiency, reducing the total carbon emissions (46, 48), and the research and application of carbon reduction technology can improve carbon efficiency (49, 50). However, Some studies believe that the promotion effect of technology on improving energy efficiency is not enough to offset the expansion effect of carbon emissions in the process of production and living, which is not conducive to the ultimate improvement of carbon emission efficiency (8). For example, Lee and Brahmasrene (51), Salahuddin et al. (52) investigate the impact of technological development on carbon emissions in nine ASEAN countries and all OECD countries, finding that the internet use significantly increases the carbon emissions in these countries. Another viewpoint is that the impact of technological innovation on carbon emission efficiency is uncertain (53). The “double-edged sword” effect of technological innovation not only enhances energy utilization efficiency, but also intensifies the increase of energy consumption and total carbon emissions. The direction of technological innovation's influence on carbon emission efficiency is unknown due to the combined action of the driving and constraining effects (54).

To sum up, there is no uniform answer to the impact of technological innovation on TCEE, which could be owing to differences in research regions, time periods and backgrounds. In addition, most studies in this field ignore the spatial effect. Although some studies do consider the spatial effect, they often assume that regional interactions are constant, ignoring the asymmetric effect caused by regional differences, which leads to biased conclusions. Therefore, our research attempts to solve the following problems: First, what is the development level of the technological innovation and TCEE in China, and is there a spatial relationship between them? Second, would technological innovation have a significant impact on TCEE? If so, which effect is more dominant: spillage or siphon? Third, is there any heterogeneity in the spatial impact of technological innovation on the TCEE? Does government intervention work? Fourth, How does technological innovation affect TCEE, and what is its transmission mechanism?

## Materials and methods

### Data

#### Measuring index system of TCEE

We used input-output data from the transport industry from 2003 to 2018 for 30 provincial administrative regions in China (excluding Taiwan, Tibet, Hong Kong, Macau). The socioeconomic data were obtained from the China Statistical Yearbook (55), while the energy data were obtained from the China Energy Statistics Yearbook (56). The specific indicator descriptions are shown in Table 1.

**Table 1:** Transport carbon emission efficiency evaluation index system.

**Indicators**	**Level 1 indicators**	**Level 2 indicators**	**Unit**
Input	Infrastructure	Total mileage of road, railway, waterway, and pipeline transportation network	10,000 kilometers
	Capital stock	Capital stock in transportation	100 million
	Labor force	Individuals employed in the transportation industry	Individuals
	Energy consumption	Energy consumption in transportation	10,000 tons of standard coal
Output	Expected output	Value added in transportation	100 million
	Unexpected output	CO2 emissions of transportation	10,000 tons

Note, the capital stock of the transport industry was calculated using the perpetual inventory method (57). The data were converted to 2003 base period prices; the added value of the transport industry was also treated. Additionally, according to the conversion coefficient of standard coal, as published in the China Energy Statistics Yearbook, all types of energy were standardized and converted to calculate the energy consumption of the transport industry. Transport carbon emission data is calculated according to Liu et al. (58).

#### Technological innovation index

At present, there is no unified standard for the calculation of technological innovation index in China. On the basis of previous studies, this paper constructs China's provincial-level comprehensive index of technological innovation from seven aspects: hardware facilities, capital investment, talent training, service intensity, technological achievements, achievement transformation and energy saving level (see Table 2). Please refer to the reference of Ma et al. (31) for the specific calculation process.

**Table 2:** The index system of technological innovation.

**Target layer**	**First-level index**	**Second-level index**	**Indicator type**	**Weight**
Technological innovation index	Hardware facilities	Penetration rate of internet	Positive	0.082
	Capital investment	Proportion of science and education expenditure to government budget expenditure	Positive	0.026
	Talent training	Number of people per 10,000 with university degree or above.	Positive	0.034
	Service intensity	Full-time equivalent of R&D personnel	Positive	0.084
	Technological achievements	Number of patent authorizations	Positive	0.267
	Achievement transformation	Trade in technology markets	Positive	0.345
	Energy saving level	Reciprocal of energy intensity	Positive	0.162

#### Mediator variables

##### Economic level (*l*n*pgdp*)

On the one hand, technological innovation has greatly changed people's life and production mode, and effectively promoted economic growth. Undoubtedly, science and technology are the primary productive forces, and technological innovation is the core power of economic development (59). On the other hand, sustained economic development will improve people's quality of life and increase transport demand. In addition, the expansion of cities and population has further stimulated the growth of transport demand, which may increase energy consumption and carbon emissions (58). However, with the change of economic growth mode to green and high-quality growth, people's consumption habits and travel modes have also changed. Public transportation has become a new fashion, and clean energy vehicles are also replacing fossil energy-fueled vehicles. This will help to reduce carbon dioxide emissions, thus affecting the changes of TCEE. Therefore, we believe that technological innovation can affect the TCEE by improving economic operation efficiency, reducing economic costs and changing travel modes. The economic level is expressed as the logarithm of GDP per capita, which is the data were converted to 2003 base period prices.

##### Transport structure

Technology innovation promotes the optimization and upgrading of transport structure in two aspects. One is the transformation of electrification. Science and technology innovation promotes the widespread application of natural gas buses and new energy vehicles. The other one is the digital upgrade. New Internet technologies is constantly integrated with the intelligent transport field to make more effective use of resources (60). For example, the Electronic Toll Collection (ETC) charging system reduces the braking and restarting of vehicles, which can reduce carbon dioxide emissions by over 50%. In terms of new infrastructure such as high-speed rail, intercity rail transit and charging pile network, the comprehensive application of artificial intelligence (AI), big data, cloud computing and other technologies can improve transport efficiency and reduce resource consumption and carbon emissions. Energy consumption mainly reflects the impact of the transport structure on the TCEE. As a “green” mode of transport, an increased proportion of railway and waterway transport use yields reduced energy consumption, which is conducive to improving the TCEE. Conversely, the higher the proportion of road transport, the lower the TCEE (19). Based on the large proportion of current road transport in China, we used the ratio of road turnover and comprehensive turnover to measure the transport structure.

#### Control variables

To make the results more accurate, some important control variables are added to the model. It include population size (ln*pop*), industrial structure (*ins*), urbanization level (*urban*), energy structure (*ens*), and transport intensity (*tri*). The following describes all of the variables used in this study.

##### Population size

The impact of the population size on the TCEE is bidirectional (19). The expansion of the population scale accelerates the spatial flow of people and goods between provinces, resulting in an increase in the transport demand, which in turn leads to an increase in energy consumption and CO2 emissions and a decrease in the TCEE. Furthermore, an increase in the transport demand due to population expansion increases the economic output of the transport industry and improves the TCEE. We used the total population of a province to determine its population size.

##### Industrial structure

The optimisation of and upgrades to the industrial structure can promote regional economic growth, increase transport demand, and change transport intensity. Furthermore, the evolution of the industrial structure can change the energy consumption structure and transition economic development from relying on fossil fuels, such as coal and petroleum, to clean energy, which effectively reduces carbon emissions and improves the TCEE (61). In this study, the proportion of the tertiary industry was used to represent the industrial structure.

##### Urbanization level

Urbanization is a dynamic process, involving population, space, economy and society. On the one hand, the advancement of urbanization can effectively promote population agglomeration and economic growth. On the other hand, urban expansion will bring more traffic demand, and increase carbon emissions of industry and service industries and product consumption. Therefore, the influence of urbanization on TCEE is uncertain. We use the ratio of urban population to the total population to express the urbanization level.

##### Energy structure

The impact of the energy structure on the TCEE mainly depends on the consumption ratio of diesel oil and gasoline. Owing to its high carbon emissions coefficient and maximum consumption, the higher the ratio of the energy structure, the lower the TCEE (20). Therefore, the energy structure was expressed as the ratio of diesel and gasoline consumption to the total energy consumption of the transport industry.

##### Transport intensity

Transport intensity can reflect the relationship between transport and economic development, which is usually expressed as the ratio of the transport turnover to the regional GDP. A lower transport intensity usually indicates a higher technical level of transport organization and management (Shao and Wang, 2021); thus, the TCEE is higher. When calculating this index, passenger and freight volumes were converted into a comprehensive conversion turnover according to the conversion coefficient specified by the Chinese statistical system.

The descriptive statistics of the variables are reported in Table 3, and we calculated the variance inflation factor (VIF) of each variable to prevent multicollinearity (Table 3). The results showed that the VIF values were all < 5, indicating that no multicollinearity was present among the variables.

**Table 3:** Descriptive statistics and multicollinearity test of the variables.

**Variable**	**Obs**.	**Mean**	**Std. Dev**.	**Min**	**Max**	**VIF**
*TCEE*	540	0.480	0.264	0.091	1	-
*S*	540	0.126	0.103	0.009	0.728	4.31
ln*pgdp*	540	10.324	0.740	8.218	12.013	4.91
*trs*	540	0.332	0.186	0.006	0.729	1.32
ln*pop*	540	8.176	0.749	6.280	9.443	1.52
*ins*	540	0.443	0.095	0.283	0.839	3.65
*urban*	540	0.532	0.149	0.238	0.942	4.25
*ens*	540	0.689	0.205	0.081	1.028	2.34
*tri*	540	0.440	0.388	0.062	3.961	1.30

### Methods

#### SBM-DEA model for TCEE calculation

The DEA model is the most popular method for measuring the carbon emissions efficiency (35, 62, 63). Among the various DEA models (e.g., the CCR, BCC, and SBM, among others), the SBM model proposed by Tone (64) considers unexpected output and reveals the influence of slacks on the measured value. Therefore, we selected the SBM model to measure the TCEE. We do not give the particular calculation formula in this work because the SBM model is mature and widely used. Please see Ma et al. (31) for details.

#### Spatial Durbin model

The spatial lag model (SLM), spatial error model (SEM), spatial autoregressive (SAR) model, and spatial Durbin model (SDM) are all classic models for characterizing spatial effects (19, 65). Among them, the SLM is mainly used to describe an endogenous interaction effect between the interpreted variables (*Y*). The SAR model mainly describes an exogenous interaction effect between the explanatory variables (*X*). The SEM mainly describes an interaction effect between the error items (ε). Finally, the SDM comprehensively considers an endogenous interaction effect among the interpreted variables (*Y*) and an exogenous interaction between the explanatory variables (*X*) and related error items (ε). Therefore, we employ SDM to assess the spatial effect of technological innovation on TCEE, as follows:


(1) $$\begin{array}{l} Y_{it}=\alpha_{0}+\rho WY_{it}+\gamma S_{it}+\beta_{k}\overset{m}{\underset{k=1}{\sum }}X_{it,k}+\gamma_{1}WS_{it}\\ \;+\lambda_{k}WX_{it,k}+\mu_{i}+\xi_{t}+\varepsilon_{it}, \end{array}$$

where *Y*_*it*_ represents the TCEE of province *i* in year *t*. *Sit* is the core explanatory variable (technological innovation index) and γ is its coefficient. *W* is a spatial weight matrix. ρ, γ1 represents the coefficient of the spatial lag term for the *Y*_*it*_ and *S*_*it*_, respectively. *X*_*it, k*_ is the *k*th control variable in period *t* in province *i*, β_*k*_ and λ_*k*_ are the regression coefficient and spatial lag coefficient of the *k*th control variable, respectively. *n* and *m* are the number of regions and control variables, respectively. μ_*i*_ and ξ_*t*_ represent the time and spatial fixed effects, respectively, and ε_*it*_ is a random error.

However, the introduction of spatial weights transformed the linear structure of the spatial econometric model into a nonlinear structure, potentially resulting in a feedback effect (66). As a result, the regression coefficient obtained by the SDM cannot fully reflect the impact of the *S*_*it*_ on *Y*_*it*_. To solve this problem, Lesage and Pace (67) transformed both the SDM into a partial derivative matrix and the regression results into direct, indirect, and total effects, which represent the average influence that the core explanatory variable (*S*_*it*_) has on explained variable (*Y*_*it*_) of local province, other provinces, and all provinces, respectively, calculated as follows:


(2) $$\begin{array}{l} Y={\left(I-\rho W\right)}^{-1}\left(\beta S+\theta WS\right)+E \end{array}$$

where *E* contains the error and constant term. The partial derivative matrix of the *S* to *Y*, can be written as follows:


(3) $$\begin{array}{l} \left[\frac{\partial Y}{\partial x_{1k}}\cdot \frac{\partial Y}{\partial s_{nk}}\right]=\left[\begin{array}{ccc} \frac{\partial y_{1}}{\partial s_{1k}} & \cdots & \frac{\partial y_{1}}{\partial s_{nk}}\\ \vdots & \ddots & \vdots \\ \frac{\partial y_{n}}{\partial s_{1k}} & \cdots & \frac{\partial y_{n}}{\partial s_{nk}} \end{array}\right]\\ \;={\left(I-\rho W\right)}^{-1}\left[\begin{array}{ccc} \beta_{k} & \omega_{12}\theta_{k} & \omega_{1n}\theta_{k}\\ \omega_{21}\theta_{k} & \beta_{k} & \omega_{2n}\theta_{k}\\ \vdots & \vdots & \vdots \\ \omega_{n1}\theta_{k} & \omega_{n2}\theta_{k} & \beta_{k} \end{array}\right] \end{array}$$

where the average value of the elements on the main diagonal is a direct effect, representing the influence of the *S* on *Y* in this province. The average value of elements on the off-diagonal line is indirect effect, representing the influence of the *S* on *Y* in other province. The sum of direct and indirect effect is the total effect.

#### Setting of spatial weights

Reasonable values for the spatial weight matrix is particularly important for determining the spatial relationship of the research objects. Therefore, we established the following three spatial weighting schemes to determine the impact of the spatial weight on the spatial relationship of the TCEE and technological innovation index.

##### Spatial weighting scheme based on spatial adjacency (*W*_1_) principle

There are two spatial adjacent weight matrices. One is based on a common vertex, i.e., two evaluation units require common points for adjacency. The other is based on common edges, i.e., given that there are common edges between two evaluation units, they can be considered adjacent. In this study, 30 provinces in China were used as evaluation units. As there were no common vertices among the different provinces, the second spatial adjacent weight matrix was selected.


(4) $$\begin{array}{l} W_{ij}=\{\begin{array}{l} 1\;\left(\text{Province }i\text{ and}j\text{ have a common boundary}\right)\\ 0\;\left(\text{Otherwise}\right) \end{array} \end{array}$$

##### Spatial weighting scheme based on spatial distance (*W*_2_) principle

There are three types of spatial distance weight matrices based on the following principles: minimum distance, polygon, and reciprocal distance. The spatial weight matrix based on the minimum distance principle uses a certain distance as the threshold value. If the distance between two provinces is less than the threshold value, they are considered adjacent and assigned a value of 1; otherwise, they are considered non-adjacent and assigned a value of 0 (68). According to the spatial weight matrix based on the polygon principle, the nearest points in space can form a specific polygon with a common boundary as its neighbor, with a value of 1; otherwise, the weight is 0. The spatial weight matrix, which is based on the reciprocal distance principle, states that the correlation between evaluation units is inversely proportional to their distance. The transport network breaks the the limit of distance between provinces. According to the distance attenuation principle, the third space distance weight matrix was selected as follows:


(5) $$\begin{array}{l} W_{ij}=\{\begin{array}{c} \frac{1}{d_{ij}}\;\left(i\neq j\right)\\ 0\;\left(i=j\right) \end{array} \end{array}$$

where *d* is the distance between the geographical centers of the provinces. This paper uses the longitude and latitude of the provincial capital city center to calculate the provincial distance.

##### An asymmetric spatial weighting scheme based on economy-distance reciprocal (*W*_3_)

The above two spatial weight matrices are symmetric matrices because the mutual influence between two evaluation units in these matrices is, by default, identical. However, due to the driving factors, such as the economic level, resource endowment, and others, the mutual influence between two regions differs, even with distance. Therefore, when calculating the spatial correlation of the TCEE and technological innovation index, setting the spatial weight as asymmetric may be more realistic. The asymmetric spatial weight matrix with the economy-distance reciprocal was introduced into the spatial correlation measurement.


(6) $$\begin{array}{l} W_{ij}=\{\begin{array}{c} {\left(\frac{G_{i}}{G_{j}}\right)}^{1/2}\times \frac{1}{d_{ij}}\text{(}i\neq j\text{)}\\ 0\text{(}i=j\text{)} \end{array} \end{array}$$

where *G*_*i*_ and *G*_*j*_ represent the GDP of provinces *i* and *j*, respectively.

#### Spatial mediating model

Based on the significance test results (α1) of model (1), a spatial mediating model is constructed by using the three-step method of mediating effect and spatial econometric model (69, 70), and the transmission mechanism of technological innovation on the TCEE is discussed.


(7) $$\begin{array}{l} M_{it}=\alpha_{0}+\gamma S_{it}+\beta_{k}\overset{m}{\underset{k=1}{\sum }}X_{it,k}+\gamma_{1}WS_{it}+\lambda_{k}WX_{it,k}+\mu_{i}\\ \;+\xi_{t}+\varepsilon_{it} \end{array}$$

(8) $$\begin{array}{l} Y_{it}=\alpha_{0}+\phi S_{it}+\phi_{1}WS_{it}+vM_{it}+v_{1}WM_{it}+\beta_{k}\overset{m}{\underset{k=1}{\sum }}X_{it,k}\\ \;+\lambda_{k}WX_{it,k}+\mu_{i}+\xi_{t}+\varepsilon_{it} \end{array}$$

Where *M*_*it*_ represents the mediating variables. If the coefficients γ, ν, ϕ are significant, the mediating variable *M*_*i*_ plays a partial mediation effect. If the coefficients λ, ν are significant and ϕ is not significant, the mediating variable *M*_*i*_ plays a full mediation effect.

## Results

### The spatio-temporal characteristics of TCEE and technological innovation index

**Figure 1:**
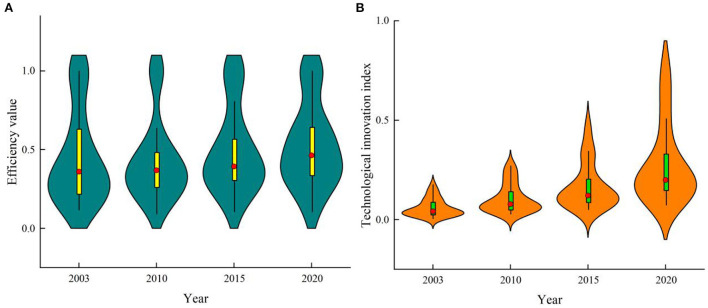
Time-varying trend of TCEE and technological innovation index in China. **(A)** Transport carbon emission efficiency. **(B)** Technological innovation index.

Figure 1 shows the time-varying trends of TCEE and technological innovation index in China. According to Figure 1A, the TCEE has obvious “double peak” distribution at four time nodes. The first peak's efficiency value is around 0.3, while the second peak's efficiency value is 1, indicating that China's transport carbon emission efficiency has obvious polarization during the study period, with most provinces having low efficiency. Specifically, from 2003 to 2010, the peak width of TCEE narrowed and the median moved up slightly, indicating that the TCEE improved. The box-plot become shorter, indicating that the degree of TCEE dispersion is reducing and the regional differences are shrinking. From 2010 to 2020, the peak width of TCEE widened, and the Kernel density curve and median moved up, which indicated that TCEE was on the rise, while the dispersion was enlarged and the polarization was aggravated. The technological innovation index shows a “single peak” distribution, as seen in Figure 1B. With the passage of time, the Kernel density curve moves up, the peak width and the box-plot stretches, indicating that China's technological innovation index presents an upward trend during the research period, but tends to be discrete and the regional differences become larger.

**Figure 2:**
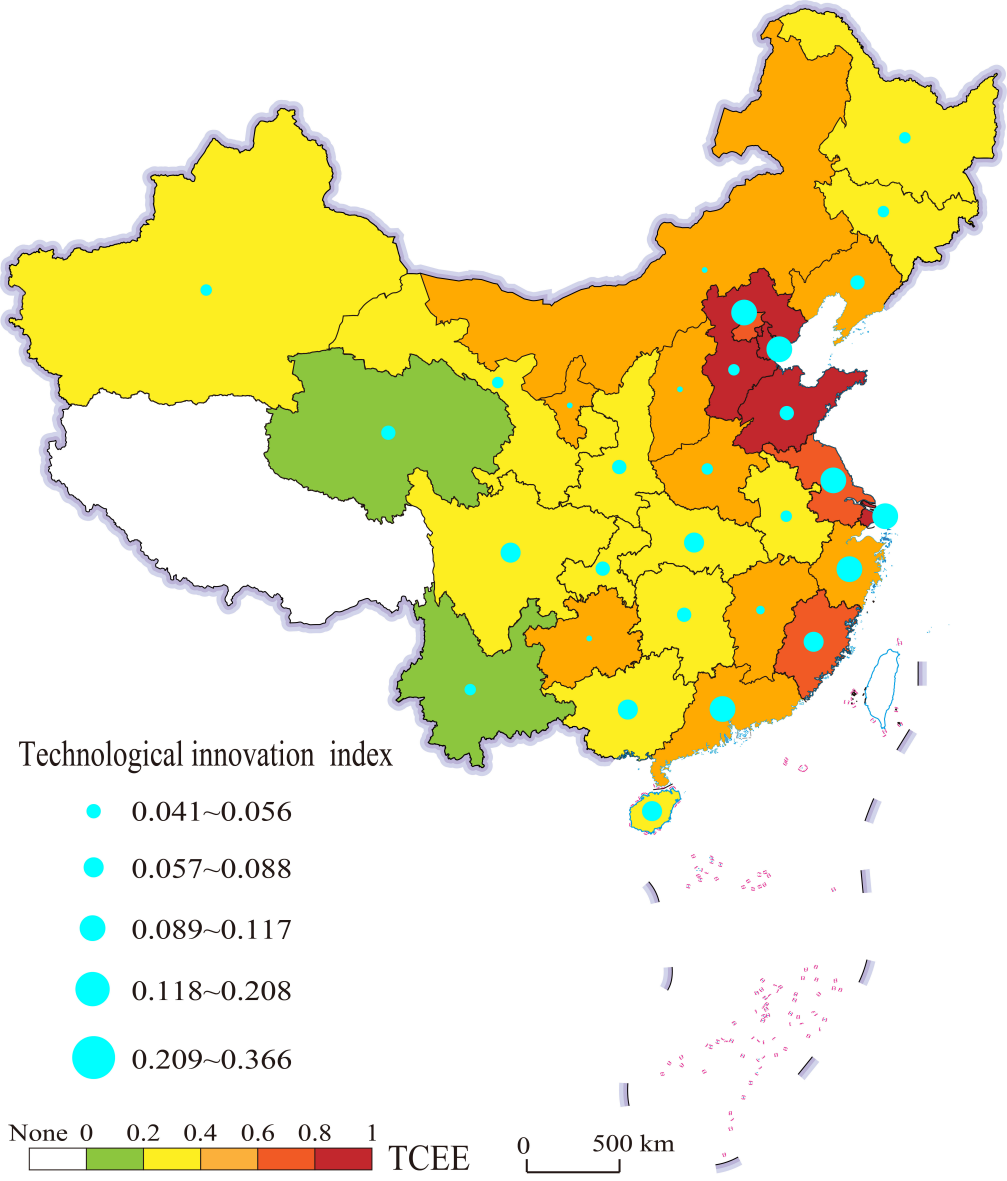
Spatial distribution pattern of TCEE and technological innovation index in China.

Figure 2 describes the spatial distribution patterns of the TCEE and technological innovation index in China. Figure 2 shows that China's technological innovation index and transport carbon emission efficiency have similar spatial distribution characteristics, namely decreasing from east to west. At the same time, the regions with high technological innovation index are mostly located in the economic circle of Beijing-Tianjin-Hebei (BTH), Yangtze River Delta (YRD) and Pearl River Delta (PRD), which is in line with the actual situation. The regions with higher TCEE include Shandong, Shanghai, Hebei, Tianjin, Jiangsu, Beijing and Fujian provinces, which are highly coincident with those with higher technological innovation index. The explanation for this could be that the eastern provinces of China are generally richer in resources, better in transport infrastructure, more advantageous in policies, and more conducive to the development of science and technology. Simultaneously, science and technology are used to promote production and improve the transport carbon emission efficiency.

### Spatial correlation test

In order to further analyze the spatial effect of TCEE and technological innovation index, we calculated Moran's *I* of TCEE and technological innovation index under three spatial weight matrices from 2003 to 2020. Please refer to Table 4 for the results.

**Table 4:** The results of spatial correlation test.

**Year**	***W***_**1**_		***W***_**2**_		***W***_**3**_	
	**Moran's *I***	***z***	**Moran's *I***	***z***	**Moran's *I***	***z***
2003	0.579***/0.348***	5.099/3.223	0.182***/0.083***	6.212/3.405	0.131***/0.067***	5.155/3.189
2004	0.572***/0.389***	5.029/3.590	0.198***/0.091***	6.654/3.682	0.154***/0.082***	5.874/3.663
2005	0.571***/0.380***	5.014/3.513	0.182***/0.088***	6.197/3.591	0.143***/0.078***	5.510/3.537
2006	0.551***/0.410***	4.890/3.763	0.165***/0.093***	5.754/3.734	0.133***/0.081***	5.239/3.631
2007	0.493***/0.382***	4.442/3.496	0.149***/0.083***	5.305/3.396	0.122***/0.071***	4.889/3.318
2008	0.394***/0.365***	3.645/3.356	0.109***/0.083***	4.217/3.406	0.093***/0.070***	4.025/3.258
2009	0.401***/0.375***	3.727/3.460	0.107***/0.082***	4.159/3.390	0.092***/0.070***	3.981/3.274
2010	0.434***/0.398***	3.988/3.614	0.115***/0.090***	4.371/3.588	0.097***/0.062***	4.145/3.015
2011	0.440***/0.379***	4.025/3.456	0.110***/0.085***	4.217/3.439	0.092***/0.060***	3.966/2.953
2012	0.429***/0.375***	3.926/3.437	0.104***/0.086***	4.059/3.471	0.086***/0.061***	3.788/2.980
2013	0.439***/0.409***	4.073/3.704	0.085***/0.093***	3.547/3.690	0.051***/0.055***	2.705/2.802
2014	0.327***/0.395***	2.985/3.606	0.061***/0.085***	2.719/3.454	0.027**/0.048***	1.925/2.576
2015	0.488***/0.396***	4.341/3.650	0.112***/0.083***	4.206/3.432	0.058***/0.051***	2.871/2.689
2016	0.561***/0.414***	4.923/3.789	0.142***/0.086***	5.030/3.510	0.079***/0.046***	3.516/2.532
2017	0.556***/0.395***	4.854/3.624	0.141***/0.078***	4.982/3.274	0.083***/0.036**	3.650/2.213
2018	0.584***/0.390***	5.117/3.605	0.160***/0.079***	5.551/3.315	0.093***/0.035**	3.975/2.174
2019	0.596***/0.376***	5.229/3.500	0.163***/0.078***	5.661/3.310	0.090***/0.032**	3.882/2.103
2020	0.603***/0.360***	5.283/3.334	0.165***/0.082***	5.691/3.415	0.092***/0.033**	3.943/2.122

TCEE, technological innovation index.

***, **, and * denote the significance at the 1%, 5%, and 10% levels, respectively.

Table 4 shows that, under three spatial weight matrices, the global Moran's *I* of China's TCEE and technological innovation index from 2003 to 2020 are all positive, and all of them passed the 5 % significance test, indicating that the TCEE and technological innovation index have a high positive spatial correlation, namely, a cluster phenomenon. The Moran's *I* calculated using the *W*_1_ and *W*_2_ spatial weight matrices were larger than that calculated using *W*_3_, showing that the spatial correlation of the symmetric spatial weight calculation was higher. However, the *p* value obtained using the symmetric spatial weight matrix changed with a change in the random Monte Carlo random test times, leading to partial uncertainty in the evaluation results. The *p* value obtained using *W*_3_ was derived under the random assumption of spatial non-correlation. Although the Moran's *I* was not high, the spatial weight matrix had a negligible influence on the *z*-value test, which showed that *W*_3_ could support the overall evaluation of the spatial correlation of the TCEE and technological innovation index. Therefore, we selects *W*_3_ as the spatial weight matrix to perform the spatial Durbin regression analysis.

**Figure 3:**
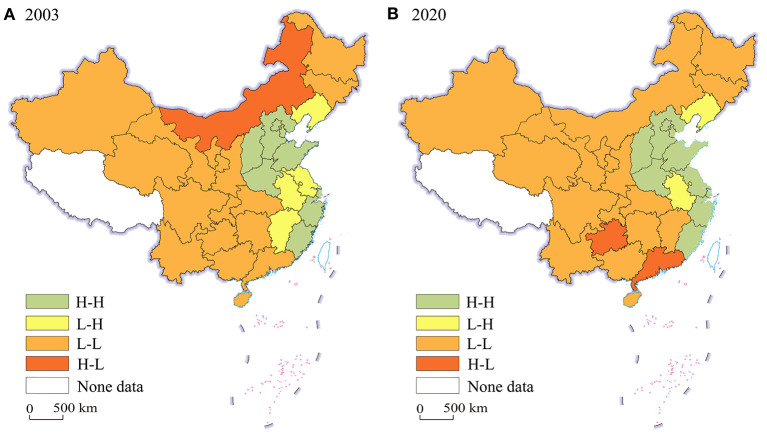
LISA agglomeration maps of TCEE. **(A)** 2003; **(B)** 2020.

**Figure 4:**
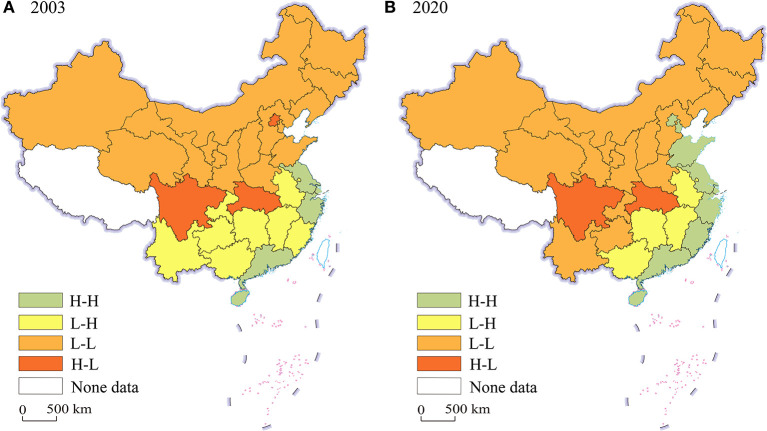
LISA agglomeration maps of technological innovation index. **(A)** 2003; **(B)** 2020.

Figures 3, 4 illustrate the LISA agglomeration maps of TCEE and technological innovation index in China, respectively. As shown in Figures 3, 4, the TCEE and technological innovation index in most provinces are positively correlated with those in the surrounding provinces since most observations belong to H-H and L-L agglomeration for both years. According to Figure 3, in 2003, 9 provinces are classified as H-H agglomeration and 16 provinces are classified as L-L agglomeration. In 2020, 10 provinces belong to H-H agglomeration, and 16 provinces belong to L-L agglomeration, accounting for 83.33 and 86.67% of the total sample, respectively. This further confirms the spatial correlation of TCEE. Spatially, the H-H agglomeration type of TCEE are relatively stable in North China and the Yangtze River Delta, while the L-L agglomeration type are mainly located in the central, western and northeast regions.

The same pattern is applicable to China's technological innovation index. Figure 4 shows the clustering results of technological innovation index. In 2003 and 2020, 5 and 8 provinces are classified as H-H aggregation type, while 14 and 15 provinces are classified as L-L aggregation type. In 2003 and 2020, the two types account for 63.33 and 76.67% of the total sample, respectively. Further, the H-H agglomeration type gradually spread from the YRD and PRD to the BR economic circle, while the L-L agglomeration type tend to spread to the south.

In a word, the spatial distribution and exploratory spatial test reveal that there is a positive spatial correlation of TCEE and technological innovation from 2003 to 2020 in China, rather than a random distribution. In addition, their geographical distribution is relatively stable, showing obvious “path dependence” characteristics. The high-value aggregation area of technological innovation index matches with the high-value aggregation area of TCEE, while the low-value aggregation area of technological innovation index matches with the low-value aggregation area of TCEE. Therefore, it may be extrapolated that technological innovation has promoted the TCEE, and the polarization of TCEE may be intensified due to the siphon effect. In order to prove this hypothesis, spatial econometric analysis is further conducted.

### Regression results of technological innovation index on TCEE

#### Parameter estimation of non-spatial panel model

Table 5 reports the linear estimation results of the technological innovation on TCEE without considering the spatial effects. Column (1) is the regression result without adding control variables. The result show that the coefficient of core explanatory variable is 0.401, which is significant at the 1% level, demonstrating that technological innovation can effectively promote the growth of TCEE. To verify the robustness of the results, we added control variables in turn to carry out stepwise regression. According to the regression results of column (2) to (6), all regression coefficients are positive and significant, indicating that technological innovation can effectively improve TCEE in China without considering the spatial effect.

**Table 5:** Basic estimation results without spatial effects.

**Variables**	**(1)**	**(2)**	**(3)**	**(4)**	**(5)**	**(6)**
*S*	0.401*** (6.49)	0.637*** (6.51)	0.587*** (5.65)	0.559*** (5.32)	0.537*** (4.91)	0.585*** (5.15)
ln*pop*		−0.282*** (−2.97)	−0.297*** (−3.11)	−0.322*** (−3.34)	−0.360*** (−3.58)	−0.445*** (−3.88)
*ins*			0.160 (1.46)	0.219* (1.90)	0.186 (1.52)	0.151 (1.21)
*urban*				−0.275 (−1.64)	−0.253 (−1.49)	−0.277 (−1.63)
*ens*					0.007 (0.13)	0.020 (0.38)
*tri*						−0.029 (−1.53)
*Cons*	0.430*** (48.33)	2.843*** (3.74)	2.954*** (3.87)	2.958*** (3.88)	3.113*** (3.98)	3.782*** (4.22)
Obs.	540	540	540	540	540	540
*R* ^2^	0.677	0.697	0.701	0.706	0.716	0.720
*Adjust*−*R*^2^	0.669	0.683	0.691	0.699	0.706	0.711

***, **, and * denote the significance at the 1%, 5%, and 10% levels, respectively; t statistics are shown between parentheses.

However, if the panel data is spatially dependent, the results of the classical econometric model may be biased, and invalid parameter estimation may be obtained because the spatial interaction between the observed data is ignored (71). As mentioned above, China's TCEE and technological innovation index have obvious spatial correlation characteristics. Therefore, we use SDM to further explore the influence of technological innovation on TCEE.

#### Model screening and testing

Before using SDM for regression, it is necessary to choose the appropriate spatial econometric model and spatial weights. We conducted a Lagrangian multiplier (LM) test (see Table 6). Among the four models, *W*_1_ failed the significance test of spatial autocorrelation error term. Furthermore, *W*_2_ did not pass the significance test of spatial lag explanatory variables and spatial auto-correlation error term. Only the LM test of *W*_3_ passed the significance test at 1 %. Therefore, this verified the spatial effects of the sample data and the robustness of *W*_3_.

**Table 6:** Lagrangian multiplier (LM) test results.

**Weights**	**Test statistics**	**No fixed**	**Time-fixed**	**Spatial-fixed**	**Spatio-temporal fixed**
*W*_1_	Lag-LM	55.7101***	3.6057*	13.6527***	5.7725**
	Lag-R-LM	12.8401***	9.3337***	17.8668***	4.6167**
	Error-LM	49.5304***	0.8725	39.3853***	3.5660**
	Error-R-LM	6.6604***	6.6004***	43.5994***	1.4101
	R^2^	0.6314	0.6502	0.8694	0.9811
*W*_2_	Lag-LM	46.4152***	0.0002	87.7985***	9.3103***
	Lag-R-LM	3.2049*	4.4000**	12.4370***	1.9326
	Error-LM	56.8292***	7.3925***	21.8372***	7.6808***
	Error-R-LM	13.6188***	11.3925***	37.4757***	0.3031
	R^2^	0.6319	0.6530	0.8675	0.9816
*W*_3_	Lag-LM	18.9134***	1.6887	73.9362***	16.6302***
	Lag-R-LM	32.6917***	8.1017***	15.9828***	11.2288***
	Error-LM	54.5495***	10.6771***	91.2727***	15.7408***
	Error-R-LM	68.3278***	9.0902***	33.3193***	10.3394***
	R^2^	0.6318	0.6539	0.8676	0.9821

***, **, and * denote the significance at the 1%, 5%, and 10% levels, respectively.

We note that the Lag-LM under the time-fixed effect failed the significance test (1.6887) in the LM test with *W*_3_ as the spatial weight matrix. Therefore, caution should be exercised when choosing this model. In this paper, the Ward test, likelihood ratio (LR) test, and Hausman test were used to select the model and determine whether the SDM would degenerate into an SLM or SEM (see Table 7). The test results showed that the Lag-Ward, Lag-LR, Error-Ward, and Error-LR tests were all significant at the 1 % level, indicating that the SDM should be used. Furthermore, the Hausman test results showed that the model rejected the random effect (45.1509^***^). The model *R*^2^ was the largest (0.9821) under the spatio-temporal fixed effect, as reported in Table 5. Therefore, the SDM with fixed time and space was selected as the optimisation model for the regression analysis.

**Table 7:** Spatial model test results.

**Test statistics**	***W*_1_**	***W*_2_**	***W*_3_**
Lag-Ward	18.0367**	37.0135***	39.3284***
Lag-LR	17.3539**	35.7360***	37.4137***
Error-Ward	17.4099**	33.7804***	38.5969***
Error-LR	15.2676*	15.9652**	36.0248***
Hausman	39.9577***	80.9123***	45.1509***

***, **, and * denote the significance at the 1%, 5%, and 10% levels, respectively.

#### Parameter estimates from spatial Durbin model

Table 8 presents the regression results for the SDM. The coefficient, ρ, was −1.3143, which passed the 1 % significance level test, indicating that there was a notable negative spatial spillover effect on the TCEE in China, i.e. siphon effect. The *R*^2^ was 0.7627, which indicates that the regression result of the SDM is good. As mentioned above, whether technological innovation can improve TCEE in the surrounding provinces depends on the trade-off between “spillover effect” and “siphon effect”. If the former exceeds the latter, i.e., technological innovation can promote TCEE in the surrounding provinces, the coefficient of W^*^S should be positive, and vice versa. Table 8 shows that the W^*^S is significantly negative, indicating that technological innovation has reduced the TCEE of the surrounding provinces. Specifically, the “siphon effect” of local attraction of labor, capital and other elements is greater than the “spillover effect” of technological innovation on the flow of green production technology, environmental awareness and advanced systems to the surrounding provinces.

**Table 8:** Regression results of spatial Durbin model.

**Variables**	**Coefficient**	***t***	**Variables**	**Coefficient**	***t***
*S*	0.4379***	4.18	W*S	−0.0941**	−2.03
ln*pop*	−0.0020	−0.02	W*ln*pop*	0.1418	0.13
*ins*	0.1217**	1.97	W*ins	−3.5613**	−2.23
*urban*	−0.4699***	−2.90	W*urban	2.7768**	2.09
*ens*	−0.1018**	−2.09	W*ens	−0.1371	−0.36
*tri*	−0.0101	−0.60	W*tri	0.7493***	3.30
ρ	−1.3143***	−6.28	*R* ^2^	0.7627	
σ^2^	0.0057***	16.49	Log-likelihood	595.1071	

***, **, and * denote the significance at the 1%, 5%, and 10% levels, respectively.

Control variables, the coefficients of industrial structure (*ins*) are significantly positive, indicating that the factor have positive promotion effects on TCEE. Urbanization level (*urban*) and energy structure (*ens*) coefficient are negative, and significant at 1%, and 5% levels, respectively, indicating that they have a negative impact on TCEE. This is consistent with Zhao et al. (19). Transport intensity (*tri*) has a negative impact on the TCEE, Transport intensity reflects the relationship between transport and economic growth. The reduction in transport intensity means a reduction in turnover per unit GDP. Therefore, transport intensity has a negative impact on TCEE through economic growth; that is, the transport intensity decreases and carbon emission efficiency improves. In addition, the lag items of industrial structure, urbanization level, energy structure and transport intensity have a significant impact on TCEE, among which the coefficient of urbanization level and transport intensity are significantly positive, while the industrial and energy structure are significantly negative.

Because of the feedback effect, the regression coefficient of SDM can not fully reflect the influence of core explanatory variables on TCEE. Therefore, the direct, indirect, and total effects are used to reflect the influence of technological innovation on the TCEE (see Table 9). The direct effect of the technological innovation index is positive and the indirect effect is negative, both of them are significant, indicating that technological innovation can improve the local TCEE, but reduce the TCEE of the surrounding provinces through siphon effect. In addition, the total effect of the technological innovation index is significantly positive, indicating that technological innovation can improve TCEE on the whole. Therefore, local governments should strive to break down the regional barriers, speed up the regional flow of technological elements, and transform the siphon effect of technological innovation on TCEE into a spillover effect, so as to improve the TCEE as a whole.

**Table 9:** Direct, indirect and total effects of technological innovation on TCEE.

**Variables**	**Direct effect**		**Indirect effect**		**Total effect**	
	**Coefficient**	***t***	**Coefficient**	***t***	**Coefficient**	***t***
*S*	0.5357***	4.99	−0.2695***	−2.86	0.2662*	1.74
ln*pop*	0.0021	0.02	0.0633	0.12	0.0655	0.14
*ins*	0.0032	0.02	−1.6372**	−2.21	−1.6340**	−2.09
*urban*	−0.5904***	−3.22	1.5609**	2.53	0.9705*	1.68
*ens*	−0.1001*	−1.85	−0.0112	−0.06	−0.1113*	−1.66
*tri*	−0.0393**	−2.30	0.3574***	3.21	0.3181***	2.94

***, **, and * denote the significance at the 1%, 5%, and 10% levels, respectively.

### Results robustness test

#### Robustness test of control variables lag

Robustness testing of results is essential. First of all, we deal with all the control variables with lag one period, which aims at eliminating the interference of the control variables on TCEE (72). The results of robustness test show that the direct and total effect coefficient of technological innovation index are significantly positive, while the indirect effect coefficient is negative and significant (see Table 10), which indicates that technological innovation can effectively improve TCEE in the province, but it is not conducive to neighboring provinces. The regression results of control variables are basically consistent with Table 9, and the previous conclusion is still valid.

**Table 10:** Robustness test results of control variables lag.

**Variables**	**Direct effect**		**Indirect effect**		**Total effect**	
	**Coefficient**	***t***	**Coefficient**	***t***	**Coefficient**	***t***
*S*	0.5320***	4.86	−0.2637***	−2.85	0.2683*	1.74
ln*pop*	0.0400	0.31	−0.3932	−0.74	−0.3532	−0.74
*ins*	0.0613	0.33	−1.3440*	−1.80	−1.2826*	−1.62
*urban*	−0.7405***	−3.71	1.9927***	2.89	1.2522**	2.03
*ens*	−0.0668	−1.14	0.0650	0.33	−0.0018	−0.01
*tri*	−0.0564***	−3.23	0.4037***	3.48	0.3474***	3.06

***, **, and * denote the significance at the 1%, 5%, and 10% levels, respectively.

#### Robustness test of spatial weight matrix

Considering that the estimation coefficients of the spatial econometric model is sensitive to the spatial weight matrix, we use adjacency matrix (*W*_1_) and distance matrix (*W*_2_) to analyze the robustness of the results. The results show that changing the spatial weight matrix has little influence on the estimation results, which once again proves that our conclusion is reliable (see Supplementary Table 1).

#### Robustness test by adjusting the time bandwidth

We continue to perform robustness testing by adjusting the time bandwidth to eliminate non-common trend issues due to the excessively long time span. After deleting the data before 2005, 2007 and 2010 respectively, the result of the core explanatory variables is basically consistent with the regression results in Table 8 after shortening the time bandwidth (see Supplementary Table 2).

#### Robustness test of random sampling

Finally, in order to avoid the chance of results, we used the random sampling method to regress the samples (73, 74). Because the spatial econometric regression model requires the panel data to be balanced, this paper randomly deletes the data of 3 years and repeats it several times. Some results can be found in Supplementary Table 3. The result is the regression value of a random sample instead of the average value of several samples. The slight change in the core explanatory variables in the regression results shows that the results in Table 8 are relatively stable, and our conclusion is reliable.

### Spatial heterogeneity: The role of government intervention

According to Table 2, the weight of the achievement transformation index is 0.345, which is much larger than the weight of the indicators such as hardware facilities (0.082), capital investment (0.026), talent training (0.034) and service intensity (0.084), indicating that the level of technological innovation depends on the transformation of scientific and technological achievements, rather than factors investment. Therefore, technological innovation is a highly market-oriented factor of production. Considering the different degrees of regional market, the driving forces of transport carbon emission efficiency will be different. In China, government intervention has a significant impact on the level of regional market. Excessive government intervention may damage the regional market and further inhibit the promotion of technological innovation on TCEE. Therefore, we conducted an empirical test based on different levels of local government intervention to analyze this heterogeneous effect.

According to the classification standard of the National Bureau of Statistics, 30 provinces in China are divided into the eastern, central and western regions. The eastern region includes 11 provinces and cities, including Beijing, Tianjin, Hebei, Liaoning, Shandong, Jiangsu, Shanghai, Zhejiang, Fujian, Guangdong and Hainan. Including eight provinces in central China, such as Heilongjiang, Jilin, Shanxi, Anhui, Jiangxi, Henan, Hubei and Hunan. The other 12 provinces belong to the western region, namely, Inner Mongolia, Guangxi, Chongqing, Sichuan, Guizhou, Yunnan, Shaanxi, Gansu, Qinghai, Ningxia and Xinjiang. We use the ratio of transportation expenditure to GDP to measure the level of government intervention (*gov*). The calculation results show that the central (2.344) and western (3.079) regions of China generally have a higher level of government intervention, while it is relatively low in the east (1.048). As mentioned above, the spatial Dubin model with both time and spatial fixed is used to analyze the heterogeneity of government intervention. The results are shown in Supplementary Table 4.

According to Supplementary Table 4, the spatial autoregressive coefficient ρ of TCEE in each region is negative and significant, and its absolute value decreases from east to west, indicating that the siphon effect is stronger in regions with higher TCEE. At the national level, the coefficient of S^*^gov is significantly negative (−0.1324). This indicates that excessive government intervention will inhibit the promotion of technological innovation on TCEE. As for the eastern region, the coefficient of S^*^gov is 0.1058, which passes the significance test of 5 %. It shows that technological innovation can significantly promote the TCEE in the eastern region where government intervention is low, but it is not significant in the central region. On the contrary, the coefficient of S^*^gov in the west is negative (−0.2460), and it has passed the significance test of 1 %, which shows that excessive government intervention has hindered the improvement of TCEE by technological innovation. The above results prove that the impact of technological innovation on TCEE is spatially heterogeneous under government intervention. In addition, the influence of control variables on TCEE also has significant spatial heterogeneity. For example, the increase of population size will inhibit the increase of TCEE in the east, but it has a positive promoting effect on the west. The improvement of energy structure has a significantly higher promotion effect on TCEE in the eastern and central than in the western region.

### Mechanism analysis

#### Technological innovation, economic growth and TCEE

Supplementary Table 5 shows the test results with economic level (ln*pgdp*) as the mediator variable under the three spatial weight matrices. The results of columns (1), (4), and (7) show that technological innovation has significantly promoted the TCEE. Similarly, the results in column (2), (5), and (8) are all significantly positive, indicating that technological innovation can significantly improve the level of economic development. Columns (3), (6) and (9) show that the regression coefficients of technological innovation and economic level under the three spatial weight matrices are 0.528 and 0.364, 0.566 and 0.243, 0.445 and 0.211, respectively, which indicates that technological innovation can improve the TCEE by promoting economic growth. Both coefficients have passed the 1% significance test, indicating that there is a partial mediating effect on economic growth. Specifically, scientific and technological innovation has been deeply integrated into all aspects of social and economic development. While improving production capacity, quality and efficiency, its contribution to the national economy has increased significantly. Besides, economic growth is also related to energy consumption and carbon emissions in transport. Changes of consumption concepts and travel mode directly affect the scale and structure of energy consumption, and then affects the TCEE.

#### Technological innovation, transport structure and TCEE

Supplementary Table 6 shows the test results of transport structure (*trs*) as the mediator variable under the three spatial weight matrices. The results in columns (1), (4), and (7) are the same as those discussed above. The results in columns (2), (5) and (8) are all significantly negative, indicating that technological innovation can optimize the transport structure and significantly reduce the proportion of road transport. Columns (3), (6) and (9) show that when both technological innovation and transport structure are incorporated into the regression model, the technological innovation coefficients are 0.663, 0.622 and 0.470, and the transport structure coefficients are −0.030, −0.037 and −0.028, respectively, which indicates that technological innovation can improve the TCEE by optimizing the transport structure. Furthermore, the technological innovation coefficient has not passed the significance test, while the transport structure coefficient has passed the 1% significance test, indicating that the transport structure has a complete mediating effect. Specifically, with the development of the scientific and technological revolution, the transport field is becoming more and more green and intelligent. The deep integration of technological innovation and transport mode has accelerated the “road-to-railway” and “road-to-water” transport, and greatly optimized the transport structure. In addition, the optimization of transport structure reduces the consumption of fossil energy (such as, gasoline, diesel) and carbon emissions, which in turn affects the TCEE.

## Discussion and conclusions

### Discussion

It is a common consensus in various regions to promote carbon emission reduction and improve carbon emission efficiency through technological innovation, and many scholars have confirmed this view. Unfortunately, most researches ignore the spatial effect, which leads to inaccurate measurement results. We use the spatial econometric model to test the spatial effect of technological innovation on transport carbon emission efficiency, and identify the spatial heterogeneity of government intervention and the transmission mechanism of technological innovation on TCEE.

In Section 4.3.3, the regression coefficient of technological innovation to TCEE is significantly positive, indicating that technological innovation can effectively improve TCEE, which reminds us that we should spare no effort to improve the level of technological innovation. However, the results in Table 2 show that the weight of achievement transformation index is 0.345, and the weight of capital investment index is only 0.026, which indicates that the level of technological innovation mainly depends on the degree to which scientific and technological achievements are transformed into productive forces, that is to say, we should pay attention to the transformation of scientific and technological achievements into productive forces instead of blindly increasing scientific and technological investment, which is the key to improving the level of technological innovation.

In addition, that result in Table 8 indicate that transport carbon emission efficiency has a significant siphon effect, and technological innovation will also hinder the improvement of TCEE in the surrounding provinces. Therefore, the government should be alert to the siphon effect. We can attempt to address these issues by enhancing regional cooperation, breaking down regional barriers and encouraging the flow of scientific and technological innovation elements.

In Section 4.5, after joining the government intervention index, the spatial effect of technological innovation on TCEE shows significant spatial difference. Specifically, the regression coefficient in the eastern region is significantly positive, while that in the central and western regions is significantly negative, which indicates that excessive government intervention is not conducive to the improvement of TCEE by technological innovation. The government should streamline administration and delegate power, simplify administrative inspection and approval procedures, improve government efficiency, and minimize excessive administrative oversight of technology innovation.

Anything else, when formulating policies, the government should fully consider the regional differences, such as economy, population, industrial structure and other factors, and formulate targeted and differentiated emission reduction policies. For example, in the northeast and west, where there is a high rate of brain drain and a small population, the government should encourage childbearing, and formulate a preferential settlement policy to attract talents, in order to boost economic growth and reduce carbon emissions. Giving full recognition to the leading roles of developed provinces, such as the BR, YRD, and PRD in western China, promoting the overflow of new technologies, methods, and knowledge, reducing regional differences, and realizing overall improvements to the TCEE are all necessary.

The results in Section 4.4 show that technological innovation can improve TCEE by promoting economic growth and improving transportation structure. Economic growth is related to all aspects of social development. Improving the transportation structure can be achieved through the following two points. On the one hand, the government should vigorously develop clean energy vehicles, such as those using pure electric power, hybrid power and hydrogen energy, to reduce the consumption of fossil fuels such as gasoline and diesel. On the other hand, vigorously promote the transformation from road to water and rail transport, and adopt new transport organization methods such as multimodal transport to promote container transport and reduce transport intensity, in order to optimize the energy and transport structure, and improve TCEE.

### Conclusions

The TCEE and technology innovation index have similar spatio-temporal evolution characteristics in China, i.e. the value increases with time while the regional differences continue to expand. Spatially, the high-value provinces of TCEE and technological innovation are all distributed in the eastern region, showing a decreasing distribution from the eastern coast to the western inland.

China's TCEE and the development of technological innovation have a strong positive spatial correlation and high stability, showing an obvious “path dependence” feature. Spatially, the high-value (low-value) agglomeration area of technological innovation index is highly coincident with the high-value (low-value) agglomeration area of TCEE.

The TCEE in China has a significant siphon effect, and the higher the efficiency, the stronger the siphon effect in the region. Technological innovation can effectively improve the TCEE in local province, but it inhibits the improvement of TCEE in the surrounding provinces through siphon effect.

Spatial heterogeneity analysis reveals that excessive government intervention will inhibit the promotion of technological innovation on TCEE. The central and western regions, which have a higher level of government intervention than the eastern region, have more obvious inhibition effects.

Mechanism analysis shows that technological innovation can improve the TCEE by improving economic level and optimizing transport structure. Among them, economic growth has a partial mediating effect, and the transport structure has a full mediating effect.

## Data availability statement

The original contributions presented in the study are included in the article/Supplementary material, further inquiries can be directed to the corresponding author.

## Author contributions

Methodology: PJ. Software, validation, formal analysis, writing—original draft preparation, and visualization: QM. Writing—review and editing, supervision, and funding acquisition: PJ and HK. All authors have read and agreed to the published version of the manuscript.

## Funding

This research was funded by the National Key Research and Development Program of China (NO. 2019YFB1600400), National Natural Science Foundation of China (NO. 72174035), the Fundamental Research Funds for the Central Universities (NO. 3132022641), LiaoNing Revitalization Talents Program (NO. XLYC2008030), and the Cultivation Program for the Excellent Doctoral Dissertation of Dalian Maritime University (NO. 2022YBPY012).

## Conflict of interest

The authors declare that the research was conducted in the absence of any commercial or financial relationships that could be construed as a potential conflict of interest.

## Publisher's note

All claims expressed in this article are solely those of the authors and do not necessarily represent those of their affiliated organizations, or those of the publisher, the editors and the reviewers. Any product that may be evaluated in this article, or claim that may be made by its manufacturer, is not guaranteed or endorsed by the publisher.
