# Supplementary material for: The impact of technological innovation on transport carbon emission efficiency in China: Spillover effect or siphon effect?
Source: Front Public Health. 2022 Oct 4;10:1028501. doi: 10.3389/fpubh.2022.1028501 (PMC9577301; doi:10.3389/fpubh.2022.1028501)
Supplement: Supplementary file 1 [file Data_Sheet_1.docx]

**Supplementary Table 1. Robustness test results of spatial weight matrix.**

| *S* | *W*_1_ | | *W*_2_ | |
| --- | --- | --- | --- | --- |
|  | Coefficient | *t* | Coefficient | *t* |
| Direct effect | 0.6110^***^ | 5.20 | 0.6179^***^ | 5.49 |
| Indirect effect | -0.3166^***^ | -3.54 | -0.5289^***^ | -3.94 |
| Total effect | 0.2944^*^ | 1.84 | 0.0890^**^ | 2.43 |
| Control variables | Yes | Yes | Yes | Yes |
| Time fixed effect | Yes | Yes | Yes | Yes |
| Spatial fixed effect | Yes | Yes | Yes | Yes |
| *ρ* | -0.2866^***^ | -4.05 | -1.5693^***^ | -7.30 |
| *σ*^2^ | 0.0068^***^ | 16.25 | 0.0058^***^ | 15.42 |

Note: ^***^ *p*<0.01, ^**^ *p*<0.05, ^*^ *p*<0.1.

**Supplementary Table 2. Robustness test results by adjusting the time bandwidth.**

| *S* | （1） | | （2） | | （3） | |
| --- | --- | --- | --- | --- | --- | --- |
|  | Coefficient | *t* | Coefficient | *t* | Coefficient | *t* |
| Direct effect | 0.4741^***^ | 4.41 | 0.4427^***^ | 3.65 | 0.4176^***^ | 2.97 |
| Indirect effect | -0.3071^***^ | -2.54 | -0.3506^*^ | -1.74 | -0.3180^**^ | -1.98 |
| Total effect | 0.1670^*^ | 1.49 | 0.0921^**^ | 2.21 | 0.0996 | 0.11 |
| Control variables | Yes | Yes | Yes | Yes | Yes | Yes |
| Time fixed effect | Yes | Yes | Yes | Yes | Yes | Yes |
| Spatial fixed effect | Yes | Yes | Yes | Yes | Yes | Yes |
| *ρ* | -1.3354^***^ | -6.03 | -1.1759^***^ | -4.98 | -0.9777^***^ | -3.79 |
| *σ*^2^ | 0.0048^***^ | 15.65 | 0.0042^***^ | 14.13 | 0.0044^***^ | 13.05 |

Note: ^***^ *p*<0.01, ^**^ *p*<0.05, ^*^ *p*<0.1.

**Supplementary Table 3. Robustness test results of random sampling.**

| *S* | （1） | | （2） | | （3） | |
| --- | --- | --- | --- | --- | --- | --- |
|  | Coefficient | *t* | Coefficient | *t* | Coefficient | *t* |
| Direct effect | 0.5174^***^ | 4.75 | 0.5538^***^ | 4.98 | 0.4787^***^ | 3.97 |
| Indirect effect | -0.1847^***^ | -2.71 | -0.3073^***^ | -2.67 | -0.3350 | -1.19 |
| Total effect | 0.3327^*^ | 1.62 | 0.2465^*^ | 1.60 | 0.1437 | 1.15 |
| Control variables | Yes | Yes | Yes | Yes | Yes | Yes |
| Time fixed effect | Yes | Yes | Yes | Yes | Yes | Yes |
| Spatial fixed effect | Yes | Yes | Yes | Yes | Yes | Yes |
| *ρ* | -1.3918^***^ | -6.10 | -1.2015^***^ | -5.27 | -1.5683^***^ | -6.46 |
| *σ*^2^ | 0.0050^***^ | 14.86 | 0.0057^***^ | 14.86 | 0.0056^***^ | 14.11 |

Note: ^***^ *p*<0.01, ^**^ *p*<0.05, ^*^ *p*<0.1.

**Supplementary Table 4. Regression results of heterogeneity analysis.**

| Variables | National | | Eastern | | Central | | Western | |
| --- | --- | --- | --- | --- | --- | --- | --- | --- |
|  | Coefficient | *t* | Coefficient | *t* | Coefficient | *t* | Coefficient | *t* |
| *S* | 0.7207^***^ | 5.69 | 1.8844^***^ | 4.30 | -0.1664^***^ | -0.74 | -0.5171^*^ | -1.83 |
| ln*pop* | 0.0011 | 0.01 | -0.9466^***^ | -3.91 | -0.0262 | -0.10 | 0.4126^**^ | 2.09 |
| *ins* | -0.0078 | -0.04 | 0.7196^*^ | 1.83 | 0.6415^*^ | 1.72 | 0.2301 | 0.99 |
| *urban* | -0.5425^***^ | -3.34 | -0.5277^*^ | -1.87 | -0.4698 | -1.34 | -0.2637 | -0.64 |
| *ens* | -0.2901^*^ | -1.85 | -0.3229^***^ | -2.97 | -0.4910^***^ | -5.66 | -0.1944^***^ | -3.16 |
| *tri* | -0.0036 | -0.22 | 0.0253 | 0.82 | -0.4680^***^ | -3.63 | 0.2344^***^ | 3.16 |
| *gov* | 0.0145^***^ | 2.64 | -0.0300 | -0.75 | -0.0130 | -0.31 | 0.0255^***^ | 3.06 |
| *S***gov* | -0.1324^***^ | -3.31 | 0.1058^*^ | 1.70 | 0.3767 | 1.45 | -0.2460^***^ | -3.30 |
| *ρ* | -1.3704^***^ | -6.57 | -0.9121^***^ | -5.45 | -0.8996^***^ | -5.40 | -0.7335^***^ | -3.07 |
| *σ*^2^ | 0.0055^***^ | 16.57 | 0.0053^***^ | 10.02 | 0.0019^***^ | 8.52 | 0.0016^***^ | 9.48 |
| *R*^2^ | 0.7601 | | 0.7569 | | 0.7222 | | 0.7006 | |
| Log-likelihood | 603.8688 | | 220.7302 | | 230.9655 | | 345.3408 | |

Note: ^***^ *p*<0.01, ^**^ *p*<0.05, ^*^ *p*<0.1.

**Supplementary Table 5. The mechanism analysis of economic growth.**

| Variables | *W*_1_ | | | *W*_2_ | | | *W*_3_ | | |
| --- | --- | --- | --- | --- | --- | --- | --- | --- | --- |
|  | (1)  *TCEE* | (2)  ln*pgdp* | (3)  *TCEE* | (4)  *TCEE* | (5)  ln*pgdp* | (6)  *TCEE* | (7)  *TCEE* | (8)  ln*pgdp* | (9)  *TCEE* |
| *S* | 0.555^***^  (4.67) | 0.263^***^  (3.09) | 0.528^***^  (4.71) | 0.447^***^  (4.07) | 0.378^***^  (4.77) | 0.566^***^  (5.41) | 0.438^***^  (4.18) | 0.259^***^  (3.37) | 0.445^***^  (4.41) |
| *M* |  |  | 0.364^***^  (6.14) |  |  | 0.243^***^  (4.27) |  |  | 0.211^***^  (3.72) |
| *X* | Yes | Yes | Yes | Yes | Yes | Yes | Yes | Yes | Yes |
| *WX* | Yes | Yes | Yes | Yes | Yes | Yes | Yes | Yes | Yes |
| *σ*^2^ | 0.007^***^  (16.25) | 0.004^***^  (16.17) | 0.007^***^  (16.28) | 0.006^***^  (15.42) | 0.004^***^  (16.34) | 0.006^***^  (15.59) | 0.006^***^  (16.49) | 0.003^***^  (16.51) | 0.006^***^  (16.51) |
| *R*^2^ | 0.565 | 0.546 | 0.485 | 0.660 | 0.535 | 0.629 | 0.763 | 0.556 | 0.651 |
| Obs. | 540 | 540 | 540 | 540 | 540 | 540 | 540 | 540 | 540 |

Notes: *t* statistics in parentheses, ^***^ *p*<0.01, ^**^ *p*<0.05, ^*^ *p*<0.1.

**Supplementary Table 6. The mechanism analysis of transport structure.**

| Variables | *W*_1_ | | | *W*_2_ | | | *W*_3_ | | |
| --- | --- | --- | --- | --- | --- | --- | --- | --- | --- |
|  | (1)  *TCEE* | (2)  *trs* | (3)  *TCEE* | (4)  *TCEE* | (5)  *trs* | (6)  *TCEE* | (7)  *TCEE* | (8)  *trs* | (9)  *TCEE* |
| *S* | 0.555^***^  (4.67) | -0.094^***^  (-3.01) | 0.663  (0.71) | 0.447^***^  (4.07) | -0.087^**^  (-1.96) | 0.622  (5.41) | 0.438^***^  (4.18) | -0.118^**^  (-2.31) | 0.470  (4.74) |
| *M* |  |  | -0.030^***^  (-2.66) |  |  | -0.037^***^  (-2.70) |  |  | -0.028^*^  (-1.56) |
| *X* | Yes | Yes | Yes | Yes | Yes | Yes | Yes | Yes | Yes |
| *WX* | Yes | Yes | Yes | Yes | Yes | Yes | Yes | Yes | Yes |
| *σ*^2^ | 0.007^***^  (16.25) | 0.005^***^  (16.42) | 0.007^***^  (16.20) | 0.006^***^  (15.42) | 0.004^***^  (16.27) | 0.006^***^  (15.42) | 0.006^***^  (16.49) | 0.005^***^  (16.45) | 0.006^***^  (16.51) |
| *R*^2^ | 0.565 | 0.447 | 0.408 | 0.660 | 0.414 | 0.392 | 0.763 | 0.335 | 0.328 |
| Obs. | 540 | 540 | 540 | 540 | 540 | 540 | 540 | 540 | 540 |

Notes: *t* statistics in parentheses, ^***^ *p*<0.01, ^**^ *p*<0.05, ^*^ *p*<0.1.
